# VariVis: a visualisation toolkit for variation databases

**DOI:** 10.1186/1471-2105-9-206

**Published:** 2008-04-23

**Authors:** Timothy D Smith, Richard GH Cotton

**Affiliations:** 1 Genomic Disorders Research Centre, Carlton South, VIC 3053, Australia; 2 The University of Melbourne, Department of Medicine, Melbourne, VIC 3010, Australia

## Abstract

**Background:**

With the completion of the Human Genome Project and recent advancements in mutation detection technologies, the volume of data available on genetic variations has risen considerably. These data are stored in online variation databases and provide important clues to the cause of diseases and potential side effects or resistance to drugs. However, the data presentation techniques employed by most of these databases make them difficult to use and understand.

**Results:**

Here we present a visualisation toolkit that can be employed by online variation databases to generate graphical models of gene sequence with corresponding variations and their consequences. The VariVis software package can run on any web server capable of executing Perl CGI scripts and can interface with numerous Database Management Systems and "flat-file" data files. VariVis produces two easily understandable graphical depictions of any gene sequence and matches these with variant data. While developed with the goal of improving the utility of human variation databases, the VariVis package can be used in any variation database to enhance utilisation of, and access to, critical information.

## Background

Although there has been effort over the last few years to improve the quality of variation databases, with the Human Genome Variation Society publishing guidelines covering fields such as variant nomenclature [1], database content, structure and deployment [2], and quality control [3], much of the data contained in variation databases remains difficult to access. In a survey of locus specific databases (LSDBs) in 2002, Claustres et al. noted that only 54% of examined databases would fit minimal criteria for ease of use, only "some" depicted the distribution of variation within a gene and "few" possessed graphical displays, especially of a dynamic nature [4]. Huge sums of money have been invested in the search for the underlying genetic causes of disease, but much of that investment is wasted if anyone who desires the existing data is unable to not only access, but also understand, what is being presented [5]. Variation databases are heavily underfunded and usually run 'on the side' by researchers while pursuing their funded research interests [6]. This lack of time and money means that database curators often are unable to devote the necessary time to developing useful visualisation tools. However, the data that languish in these databases because they are so poorly presented could provide answers to the cause of and assist clinicians in diagnosing many human diseases if utilised effectively.

To encourage greater understanding and facilitate the interpretation of data presented in the numerous locus specific databases available on the internet, we have developed software that will give the curators of variation databases quick and easy access to some basic visualisation tools.

In one sense, such visualisation tools are commonplace. The UCSC Genome Browser [7] is an excellent tool for viewing sequence annotations, providing both graphical and text-based views. Users can view various annotation sets, including cDNA evidence, predicted genes and variation data [7]. Genewindow, developed by the National Cancer Institute, provides a gene-centric view of the human genome specifically designed for variation visualisation [8].

However, these tools are of limited use to LSDB curators as they do not enable them to easily utilise their own data sets, which are often more complete than the published literature [9], although the PhenCode project has gone some way to mitigate the difficulty in loading an LSDB into the UCSC Browser [10]. However, the requirement of directing users away from the website of a locus specific database in order to make use of these browsers is undesirable and negates the benefits of the gene-specific information that the majority of LSDBs offer above and beyond variation data: 52% of locus specific databases contain additional information on the diseases caused by the catalogued variants and 34% provide clinical information for both clinicians (91%), patients and their families (66%) [4]. While incorporating variation data into genome wide browsers is desirable, it does not replace the need for data visualisation tools that can be incorporated into an LSDB's own web site.

On the other hand, specialist variation database software packages such as UMD [11] and MUTbase [12] that include visualisation require curators to use a specific database schema and user interface, which may not always be practical or sufficient.

## Implementation

VariVis is a collection of Perl scripts designed to provide a basic set of visualisation tools specifically for LSDBs that works in parallel with the existing user interface and database of an LSDB and can access variation data stored in a wide variety of formats including Database Management Systems (DBMSs) such as MySQL, Oracle and PostgreSQL, through to flat-file repositories such as comma or tab delimited text files. LSDBs utilising the specialised variation database software package LOVD [13], are especially suited to incorporating VariVis into their repertoire of tools. Gene sequences and annotations can be accessed from a locally stored file in any of a large number of sequence file formats, including the FASTA, BSML and GenBank formats (see Table 1); or VariVis can be directed to automatically retrieve sequences from any of several online sequence databases, such as GenBank [14] or EMBL [15].

**Table 1:** Compatible Sequence File Formats

ABI	GenBank
ACE	KEGG
ALF	LocusLink
ASCIITree	MetaFASTA
BSML	PHD
CHADO	PIR
CTF	PLN
EMBL	Qual
EXP	Raw
FASTA	SCF
FASTQ	Tab
GAME	TIGR
GCG	ZTR

Upon each execution of the program, VariVis uses the external BioPerl modules [16] to access the sequence data and stores the nucleotide sequence and any available structural annotations. The nucleotide sequence is then automatically numbered according to the HGVS variation numbering guidelines [17]. The Perl DBI module [18] is then used to retrieve sequence variants from the database. As each variant is added, a set of hand written regular expressions are applied to the variant name in order to determine the variant type and location. These regular expressions are capable of recognising substitution, deletion, insertion, duplication, insertion-deletion (indel) and inversion events. More complex rearrangements are currently planned for inclusion in a later release.

The graphics are rendered by a dedicated module using basic HTML output to the user's browser.

## Results

VariVis is capable of producing two different types of graphical representations of the sequence and variation data provided. Both views initially display an overview of the gene structure, divided into introns and exons. Clicking on any of these divisions will display the selected intron or exon in further detail.

**Figure 1:**
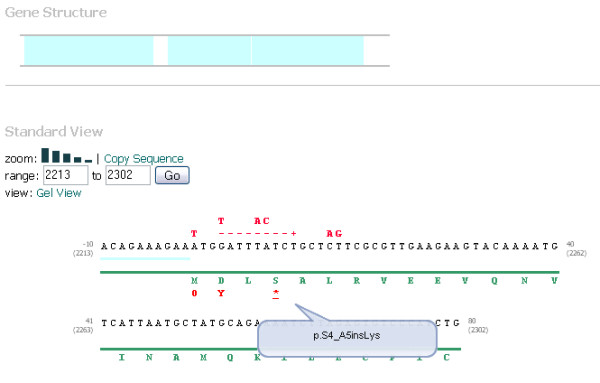
**The standard view of VariVis displays the gene sequence horizontally across the screen with nucleotide variants above the sequence track.** Annotations are displayed as coloured tracks below the sequence with the amino acid sequence below; variation within the amino acid sequence is also displayed. On-screen tools allow the user to change the magnification of the graphic, change the range being displayed or to switch between the two views.

The standard view (Figure 1) is reminiscent of traditional sequence depictions, with the sequence broken up into discrete chunks and stacked horizontally down the page. Variant nucleotides are superimposed in red above the gene sequence and their corresponding effect on the amino acid sequence in red below the amino acid sequence.

**Figure 2:**
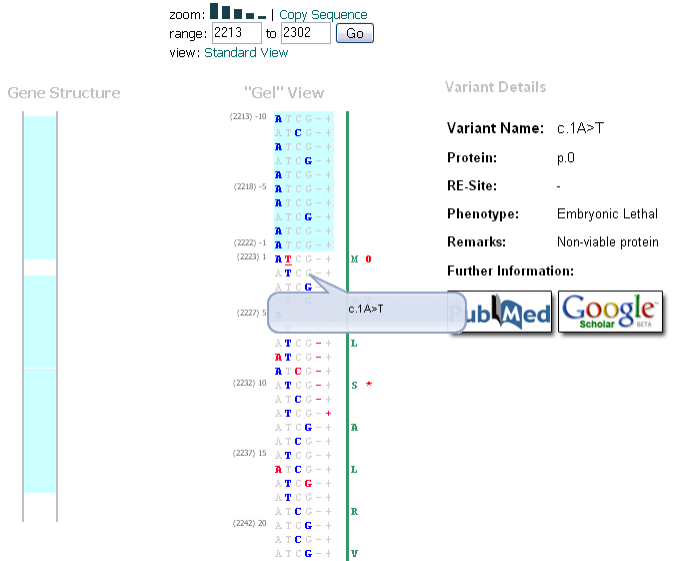
**The "Gel" View displays the gene sequence vertically, reminiscent of old sequencing gels.** Variants are highlighted in red. Clicking on a variant will display a brief summary alongside the sequence.

The second view (Figure 2), the "Gel View," has the same functionality as the first viewing option, but this time orientates the sequence vertically, allowing for an unbroken stream of data. Theoretically, it is possible that any given nucleotide in a gene can be mutated to any other nucleotide base, deleted entirely, or have an adjacent insertion. Thus, the "Gel View" displays all possible nucleotide combinations for each position, a novel strategy, highlighting the nucleotides present in the reference sequence and any variations in contrasting colours. A horizontal version of this format is also available (not shown).

In both views, the software also displays any structural annotations, such as promoter sequences and UTRs as bands of colour running in tracks alongside the gene sequence. The software also provides access to the raw sequence data, allowing users to copy or download the entire sequence, or specific chunks, negating the need to navigate to a dedicated sequence database. Clicking a variant nucleotide provides the user with a brief overview of the clinically important data present in the database for the corresponding variant, from which the user can choose to view the original report of the variant via PubMed or perform a Google Scholar search for papers discussing the variant.

## Discussion

One of the major factors we took into consideration during development of the VariVis package was ease of use, not only for the database user, but also the curator. Once the VariVis package is installed, it requires no further action on behalf of the curator, allowing them to focus entirely on collecting new data. As the graphics are generated dynamically at the time of viewing, new variants are automatically included in the displays as soon as they are added to the database. VariVis can be used to provide an overall map of a gene of interest that users can explore at their leisure, or to illustrate specific variants or features of the gene by linking directly to specific areas within the gene.

The program is easily installed by copying the source files into any directory on a web server capable of executing CGI scripts, and then configured using the provided installation script.

The study of genetic variation has and will continue to yield remarkable health benefits for all humans, and while the data currently available in online variation databases are extremely valuable, the data presentation methods employed make accessing and understanding those data very difficult. As the number of variation databases expands and more variants are discovered, the need for better presentation methods will become more apparent.

It has been argued that the amount of data that will eventually be generated from a complete catalogue of all possible genetic variants will outstrip the data generated by the Human Genome Project [9]. It is interesting to note then, that while "fancy tools" for displaying sequence data exist in the form of genome browsers such as the UCSC browser [7], very few tools currently exist for displaying sequence variation. While the variation database management software packages UMD [11] and MUTbase [12] do contain some visualisation tools, these tools are only available to the users of these particular database software packages. Visualisation tools are not available to database curators who cannot, or have chosen not to use these systems.

## Conclusion

There is a lack of visualisation tools for variation data that can be implemented on any database system. The VariVis software package is an attempt to rectify this situation by providing database curators with a visualisation tool capable of easily combining the highly curated variation data within LSDBs with sequence and annotation data regardless of their underlying database and user interface. While developed with the study of human variation in mind, the VariVis software package could be implemented in databases devoted to the cataloguing of variation within any species, particularly viruses, parasites and plants.

Very soon, the Human Variome and the myriad of health consequences it engenders will be fully described and annotated [19,20]. Novel systems need to be developed to allow fast and efficient access and use of the enormous volumes of data and information that will be generated and available, often to those less familiar with these databases. The system described here is a first step towards accomplishing efficient and useful access to these vital databases, and goes some way to satisfying recommendation D6 of the Human Variome Project [19].

## Availability and Requirements

**Project name: **VariVis

**Project home page: **

**Operating system(s): **Platform independent

**Programming language: **Perl

**Other requirements: **BioPerl and Perl DBI external modules (available via CPAN)

**License: **see 

**Any restrictions to use by non-academics: **Commercial use license can be obtained by contacting the authors

## Authors' contributions

TS carried out the programming and software design and drafted the manuscript. RC conceived of the study, and participated in its design and coordination and helped to draft the manuscript. All authors read and approved the final manuscript.
